# Exercise and eating habits among urban adolescents: a cross-sectional study in Kolkata, India

**DOI:** 10.1186/s12889-017-4390-9

**Published:** 2017-05-18

**Authors:** Soumitra Kumar, Saumitra Ray, Debabrata Roy, Kajal Ganguly, Sibananda Dutta, Tanmay Mahapatra, Sanchita Mahapatra, Kinnori Gupta, Kaushik Chakraborty, Mrinal Kanti Das, Santanu Guha, Pradip K. Deb, Amal K. Banerjee

**Affiliations:** 1 Department of Cardiology, Vivekananda Institute of Medical Sciences, Kolkata, West Bengal 700026 India; 2 Department of Cardiology, Rabindranath Tagore International Institute of Cardiac Sciences, Kolkata, West Bengal 700099 India; 3 Department of Cardiology, Nilratan Sircar Medical College and Hospital, Kolkata, West Bengal 700014 India; 4 Department of Cardiology, Institute of Post-Graduate Medical Education and Research, Kolkata, West Bengal 700020 India; 5 Fielding School of Public Health, University of California - Los Angeles, Los Angeles, CA 90095 USA; 6 Mission Arogya Health and Information Technology Research Foundation, Kolkata, West Bengal 700010 India; 7 Medica Institute of Cardiac Sciences, Medica Super Specialty Hospital, Kolkata, West Bengal 700099 India; 8 Barrackpore Population Health Research Foundation, Kolkata, West Bengal 700123 India; 9 The BM Birla Heart Research Centre, Kolkata, West Bengal 700027 India; 10 Medical College and Hospital, Kolkata, West Bengal 700073 India; 11 Charnock Hospitals Private Limited, Kolkata, West Bengal 700157 India; 12 Fortis Hospitals Private Limited, Kolkata, West Bengal 700127 India; 13 Mission Arogya Health and Information Technology Research Foundation﻿, 8 Dr. Ashutosh Sastri Road, Kolkata, West Bengal 700010 India

**Keywords:** Eating habits, Exercise, Adolescent, Urban, Kolkata

## Abstract

**Background:**

Unhealthy eating and lack of exercise during adolescence culminated into earlier onset and increasing burden of atherosclerotic cardiovascular diseases (CVDs) worldwide. Among urban Indian adolescents, prevalence of these risk factors of CVD seemed to be high, but data regarding their pattern and predictors was limited. To address this dearth of information, a survey was conducted among urban adolescent school-students in Kolkata, a highly populated metro city in eastern India.

**Methods:**

During January–June, 2014, 1755 students of 9th-grade were recruited through cluster (schools) random sampling. Informed consents from parents and assents from adolescents were collected. Information on socio-demographics, CVD-related knowledge and perception along with eating and exercise patterns were collected with an internally validated structured questionnaire. Descriptive and regression analyses were performed in SAS-9.3.2.

**Results:**

Among 1652 participants (response rate = 94.1%), about 44% had poor overall knowledge about CVD, 24% perceived themselves as overweight and 60% considered their general health as good. Only 18% perceived their future CVD-risk and 29% were engaged in regular moderate-to-vigorous exercise. While 55% skipped meals regularly, 90% frequently consumed street-foods and 54% demonstrated overall poor eating habits.

Males were more likely to engage in moderate-to-vigorous exercise [adjusted odds ratio (AOR) = 3.40(95% confidence interval = 2.55–4.54)] while students of higher SES were less likely [AOR = 0.59(0.37–0.94)]. Males and those having good CVD-related knowledge were more likely to exercise at least 1 h/day [AOR = 7.77(4.61–13.07) and 2.90(1.46–5.78) respectively].

Those who perceived their future CVD-risk, skipped meals more [2.04(1.28–3.25)] while Males skipped them less [AOR = 0.62(0.42–0.93)]. Subjects from middle class ate street-foods less frequently [AOR = 0.45(0.24–0.85)]. Relatively older students and those belonging to higher SES were less likely to demonstrate good eating habits [AOR = 0.70(0.56–0.89) and 0.23(0.11–0.47) respectively]. A large knowledge-practice gap was evident as students with good CVD-related knowledge were less likely to have good eating habits [AOR = 0.55(0.32–0.94)].

**Conclusions:**

CVD-related knowledge as well as eating and exercise habits were quite poor among adolescent school-students of Kolkata. Additionally, there was a large knowledge-practice gap. Multi-component educational interventions targeting behavioral betterment seemed necessary for these adolescents to improve their CVD-related knowledge, along with appropriate translation of knowledge into exercise and eating practices to minimize future risk of CVDs.

## Background

Exponential increase in the global burden of non-communicable diseases (NCDs) marked the beginning of the twenty-first century. Population aging, improved survival opportunities and rapid rise in obesogenic environment were the potential contributors. Cardiovascular diseases (CVDs), globally the most common cause of NCD-related deaths [1], attributed for about 17.5 million deaths in 2012 [2]. About 75% of these CVD deaths were from low-and-middle-income countries [2].

Compared to Western populations, in South Asian countries, higher prevalence and a decade earlier onset of CVDs were experienced owing to unique genetic predisposition and earlier exposure to risk factors [3, 4]. Ischemic heart disease (IHD) continued to be the most common cause of mortality among working adults (15–69 years) in Asian countries [5]. Among all major ethnic groups in South Asia, Indians were found to be at highest risk for CVDs, especially premature coronary heart diseases [4, 6, 7]. Interplay of unhealthy diet (added high sugar and refined grains), vitamin-D deficiency, tobacco use and physical inactivity contributed to this elevated risk for CVDs among Indians, especially in urban areas. In addition, the rising epidemic of type-2 diabetes mellitus further increased the vulnerability of Asian Indians to IHD [8, 9].

Thus a clear understanding of the potential predictors of CVDs appeared to be crucial for appropriate designing and timely (before the initiation of atherosclerosis) implementation of preventive interventions to control the rising tide of CVDs [10, 11]. Clustering of cardiovascular risk factors and initiation (appearance of fatty streaks) of atherosclerotic CVDs start in the second decade of life and get influenced by genetic and environmental exposures (serum lipid concentrations/smoking/obesity/hyperglycemia) during lifetime [12–14]. Furthermore, sustained high blood pressure was observed to accelerate atherosclerosis in the third decade of life [12, 15–17]. Rapid urbanization probably exposed individuals to these life-threatening yet modifiable/reversible risk factors quite early in life [11, 18]. For example, worldwide, among school-aged children, 10% were overweight [19] and >3% children and adolescents were hypertensive [20]. In addition, inadequate knowledge regarding CVDs coupled with low-risk perceptions among adolescents further heightened their susceptibility for CVDs [21].

Alike Western countries, South Asian children were also at high risk of developing CVDs in their future life, mostly because of deleterious life-styles and behaviors [22]. Among urban Indians, exposure to multiple risk factors of CVD was evidenced during adolescence and dramatically increased by 30–39 years of age [23]. Among Indian school children, high prevalence of overweight (14.4%), obesity (2.8%), sustained high blood pressure [24, 25], coupled with maternal and fetal under-nutrition were suspected to increase the future risk of CVDs [13].

Quality data regarding exercise and eating habits along with related knowledge, perceptions and consequent practices related to the risk of future CVDs in this target population were limited in India. The aim of this study was thus to assess the CVD-related knowledge, health perceptions (especially about future CVD-risk), eating habits, exercise patterns and their interplay among urban adolescent students in Kolkata, a metropolitan city in eastern India.

## Methods

### Design

A cross-sectional study was conducted among adolescent school students of Kolkata between January and December 2014. Students of 9th grade (aged 14–16 years) were selected as the study population group, as a proxy for the adolescent school-children of metropolitan Kolkata.

### Sample size and sampling strategy

Cluster random sampling strategy was used for the study using schools as the clusters. The rate of homogeneity (roh) between clusters was assumed to be equal to the intra-cluster correlations owing to the single-stage sampling method [26, 27]. Considering the possibility that characteristics relevant to this study (socio-demographics, CVD related knowledge and perception, exercise patterns and eating habits) were likely to be more correlated among the students within the same schools, we empirically assumed roh = 0.2, a relatively higher value, as per standard recommendations [26, 28].

Based on the information collected from all the governing bodies of the operational educational boards in Kolkata, the average number of students studying in the 9th grade per school was found to be approximately 75. Hence assuming the average cluster size (b) to be 75, using the formula: D = 1 + (b-1)roh, the design effect, D was calculated to be = 13.8 [26–28].

Appropriate population parameter was not available from the study area. Hence following the most conservative approach to ensure the recruitment of adequate subjects for determining the estimates with 95% confidence interval having a standard error of 0.05 (s), we empirically used 0.5 as the expected proportion (p).

Using this design effect and other assumptions as mentioned before, according to the appropriate formula [required sample size, n = p(1-p)D/ s^2^], 1580 students were required to be recruited from 21 schools [no. of clusters (schools), c = p(1-p)D/s^2^b = 21.067]. Assuming 10% non-response we planned to invite 1755 students and their guardians to participate in our study.

### Selection of schools

Initially, an exhaustive list of 426 secondary-level schools in Kolkata metropolitan area was prepared. The schools were stratified according to the socio-economic strata (higher/middle/low) they generally catered as well as the type of students enrolled (co-educational/boys only/girls only). Ensuring the recruitment of at least two schools per strata, using stratified random sampling based on school types and socio-economic status (SES) with probability proportion to size, 21 schools were selected from the list. With administrative support from the Department of School Education, Government of West Bengal, all these schools were invited. Time, date and venue of data collection were finalized ensuring maximum attendance, after completion of necessary formalities in 19 schools who agreed to participate. All students of the selected schools and their parents (preferably mothers) were invited through written letters to participate in the study.

### Study population

All 9th grade students present on the day of data collection in the selected schools, were recruited for the study if accompanied by one of their guardians (preferably mothers), agreed to participate by providing written voluntary assents and their accompanying guardians or their legally authorized representative provided written informed consents. Students with any medical or psychiatric illnesses preventing normal communications were excluded from the study.

### Data collection and measures

Information was collected through a self-administered, structured questionnaire, which was pre-tested for internal consistency and internally validated [29] in a sample of 160 students (approximately 10% of the total sample size) of same grade, recruited from two randomly selected schools of the study area. Collected socio-demographic information included: age, gender and SES of the students (based on family income).

CVD-related knowledge was assessed in the following five domains: CVDs in general (heard of heart attacks/causes/symptoms), high blood pressure (cause/symptoms), risk factors for CVDs (smoking/obesity/low cholesterol/raised sugar/stress/high fiber diet/positive family history/physical activity), prevention of CVDs and healthy nutritional habits. Based on standard textbooks and guidelines, correct and incorrect responses to individual knowledge-assessment questions were scored as 1 and 0 respectively. To estimate domain-specific and overall knowledge related to CVDs, the corresponding and overall scores were respectively summed up, log-transformed and categorized based on tertile distributions (lower = poor/middle = average/upper = good level of knowledge).

Self-perceptions regarding body-weight (normal/underweight/overweight), future risk for CVDs (yes/no) and overall health (poor/average/good) were also assessed.

To understand the exercise habit, type of activity and corresponding duration were enquired. Based on guideline of World Health Organization (WHO) for adolescent health [30], moderate to vigorous activity for at least 60 min/day on average was considered as adequate exercise.

On the other hand, to elicit the eating habit and related behavior, frequency of major meals/day (1–2/3 />3 times), frequency of snacking (≤3/4/>4 times), history of skipping meals (never/sometimes/often) and history of eating outside home (never/sometimes/often) were recorded. Average frequency of food intake for five times (including meals and snacks) was regarded as approximately appropriate as per the standard recommendations [31, 32]. Overall eating habit was determined by scoring the relatively poorer eating habits (captured based on inappropriate frequency of intake, skipping meals and eating fast foods) [31–34], in descending order and then after log-transformation of the scores, categorization as per the tertile distribution similarly as knowledge.

### Statistical analysis

Descriptive analyses of the study variables were performed to determine the mean (for age) and proportions (for categorical variables) with corresponding 95% confidence intervals (CI). Bivariate and multiple (adjusting for age, gender, SES and family history) regression analyses were performed next to measure the associations [odds ratio (OR) for bivariate and adjusted odds ratios (AOR) for multiple logistic regressions] of socio-demographic characteristics/knowledge/perceptions with exercise habit and dietary practices as well as between knowledge and perceptions regarding CVDs. Multinomial logistic regression was used when outcome variables having more than two categories. SAS version 9.3.2 was used for all statistical analyses.

## Results

Of 1755 invited students, 1652 did participate in our study (response rate of 94.1%). The mean age was 14.2 years. Proportion of females was slightly higher than males. Nearly two-third of the participants belonged to middle class families. Positive family history of CVDs was reported by about one-fifth of the students. While the majority had poor overall knowledge regarding CVDs and their preventions, a small proportion was found to have good knowledge. Domain-wise, the majority of the students had poor knowledge about CVDs, their risk factors, prevention, high blood pressure and related abnormalities as well as healthy nutritional habits. More than half of the students perceived their body weight as normal while less than a quarter perceived themselves as overweight. Less than a third of the interviewed subjects were engaged in regular moderate-to-vigorous exercise and adequate (at least 1 h) daily physically activity. Regarding dietary history, frequent snacking (four times or more/day) and eating street-foods (in the last week) were quite common (reported by more than half and one third respectively). On the other hand, consumption of more than three major meals/day and frequent skipping of meals were reported by more than 10%. Based on their dietary history, more than half of participants were found to have overall poor eating-habits (Table 1).

**Table 1:** Distribution of the study variables among participating adolescent school-students of Kolkata (*N* = 1652)

Continuous variables		Number		Mean (95%CI^a^)
Age in years		1652		14.2(14.1–14.2)
Categorical variables		Categories	Number	Percentage (95%CI)
Other socio-demographic factors	Gender of student	Female	885	53.6(51.2–56.0)
		Male	767	46.4(44.0–48.8)
	Socio-economic status	Lower	276	17.0(15.2–18.8)
		Middle	1012	62.3(60.0–64.7)
		Upper	336	20.7(18.7–22.7)
	Family history of death due to CVD^c^	No	854	78.7(76.3–81.2)
		Yes	231	21.3(18.9–23.7)
Level of knowledge regarding	CVD in general	Poor	677	41.0(38.6–43.4)
		Average	603	36.5(34.2–38.8)
		Good	372	22.5(20.5–24.5)
	Blood pressure and its abnormalities	Poor	965	58.4(56.0–60.8)
		Average	499	30.2(28.0–32.4)
		Good	188	11.4(9.9–12.9)
	Risk factors for CVDs	Poor	662	40.1(37.7–42.4)
		Average	607	36.7(34.4–39.1)
		Good	383	23.2(21.2–25.2)
	Prevention of CVDs	Poor	856	51.8(49.4–54.2)
		Average	565	34.2(31.9–36.5)
		Good	231	14.0(12.3–15.7)
	Healthy nutritional habits	Poor	847	51.3(48.9–53.7)
		Average	524	31.7(29.5–34.0)
		Good	281	17.0(15.2–18.8)
	CVD and their prevention (Overall)	Poor	719	43.5(41.1–45.9)
		Average	647	39.2(36.8–41.5)
		Good	286	17.3(15.5–19.1)
Self-perception about	Own body size	Normal	961	58.7(56.4–61.1)
		Underweight	288	17.6(15.8–19.5)
		Overweight	387	23.7(21.6–25.7)
		Obese	-	-
	Own future risk of CVDs	No	1324	81.8(79.9–83.7)
		Yes	295	18.2(16.3–20.1)
	Own overall health	Good	993	60.5(58.1–62.8)
		Average	573	34.9(32.6–37.2)
		Poor	76	4.63(3.61–5.65)
Exercise	Moderate and Vigorous Activity	No	1171	71.0(68.8–73.2)
		Yes	479	29.0(26.8–31.2)
	Exercise	No Exercise	197	12.0(10.4–13.6)
		Inadequate	965	58.8(56.4–61.2)
		Adequate	479	29.2(27.0–31.4)
Dietary history	Meals taken/day	One/two	867	52.6(50.2–55.0)
		Three	596	36.1(33.8–38.5)
		> Three	186	11.3(9.8–12.8)
	Snacks taken/day	≤ Three	806	48.9(46.5–51.4)
		Four times	690	41.9(39.5–44.3)
		> Four times	151	9.2(7.8–10.6)
	Average frequency of food intake	Approximately appropriate	785	47.6(45.2–50.0)
		More than appropriate	632	38.3(36.0–40.7)
		Much more than appropriate	232	14.1(12.4–15.8)
	Skipping meals	Never	736	44.8(42.4–47.2)
		Sometimes	690	42.0(39.6–44.4)
		Often	216	13.2(11.5–14.8)
	Eating food in the street-shop/restaurant	Never	161	9.9(8.4–11.3)
		Sometimes	870	53.3(50.9–55.7)
		Often	601	36.8(34.5–39.2)
	Overall eating habit	Poor	888	53.(51.4–56.2)
		Average	472	28.6(26.4–30.8)
		Good	292	17.7(15.8–19.5)

^a^ *CI* Confidence intervals

^b^ *SES* Socio-economic status of the families of the students

^c^ *CVD* Cardio-vascular diseases

Better knowledge (compared to poor, average or good knowledge) regarding CVDs in general, its risk factors, prevention and healthy eating among these urban adolescent school-goers was associated with having the perception regarding future risk of CVDs [for good knowledge respective Adjusted Odds Ratio, AORs were: 1.59(1.05–2.39), 2.51(1.64–3.85), 2.13(1.35–3.35) and 1.85(1.22–2.81)] (Table 2).

**Table 2:** Association between CVD ^a^ -related knowledge and perception among participating adolescent school-students of Kolkata (*N* = 1652)

Level of knowledge regarding	Categories	Perception									
		Regarding body size (Reference = Normal)				Regarding future risk of CVD (Reference = No)		Regarding general health (Reference = Good)			
		Underweight		Overweight		Yes		Average		Poor	
		AOR ^b^ (95%CI ^c^)	*p* Value	AOR ^b^ (95%CI ^c^)	*p* Value	AOR ^b^ (95%CI ^c^)	*p* Value	AOR ^b^ (95%CI ^c^)	*p* Value	AOR ^b^ (95%CI ^c^)	*p* Value
CVD (Reference = Poor)	Average	1.44(0.96–2.17)	0.077	1.25(0.89–1.76)	0.206	1.35(0.92–1.97)	0.124	0.96(0.71–1.30)	0.780	1.13(0.58–2.18)	0.717
	Good	1.20(0.75–1.90)	0.449	1.15(0.79–1.69)	0.462	**1.59(1.05–2.39)**	**0.027**	0.90(0.64–1.27)	0.557	0.57(0.23–1.39)	0.216
Blood pressure and its abnormalities (Reference = Poor)	Average	1.12(0.76–1.65)	0.560	0.99(0.71–1.37)	0.946	1.14(0.80–1.62)	0.483	**0.66(0.49–0.89)**	**0.006**	0.61(0.30–1.24)	0.171
	Good	1.18(0.68–2.06)	0.552	1.09(0.68–1.73)	0.733	1.39(0.86–2.26)	0.181	0.77(0.50–1.18)	0.226	0.77(0.28–2.08)	0.604
Risk factors for CVD (Reference = Poor)	Average	0.94(0.62–1.42)	0.780	1.16(0.82–1.64)	0.407	**2.06(1.37–3.11)**	**0.001**	1.04(0.76–1.42)	0.799	1.20(0.60–2.42)	0.605
	Good	1.28(0.81–2.03)	0.293	1.30(0.89–1.91)	0.179	**2.51(1.64–3.85)**	**<0.001**	1.26(0.89–1.78)	0.188	0.99(0.44–2.24)	0.983
Prevention of CVD (Reference = Poor)	Average	1.40(0.95–2.07)	0.093	1.25(0.90–1.73)	0.184	**1.83(1.27–2.63)**	**0.001**	1.05(0.78–1.42)	0.727	1.35(0.68–2.65)	0.391
	Good	0.97(0.56–1.69)	0.918	0.80(0.50–1.26)	0.336	**2.13(1.35–3.35)**	**0.001**	1.04(0.70–1.56)	0.839	1.40(0.58–3.37)	0.458
Healthy nutritional habits (Reference = Poor)	Average	1.05(0.70–1.59)	0.804	1.16(0.82–1.63)	0.399	1.31(0.90–1.90)	0.164	0.93(0.69–1.26)	0.644	1.30(0.64–2.65)	0.468
	Good	1.02(0.62–1.68)	0.940	1.07(0.71–1.61)	0.757	**1.85(1.22–2.81)**	**0.004**	0.78(0.53–1.14)	0.192	1.45(0.65–3.21)	0.365
CVD and their prevention (Overall) (Reference = Poor)	Average	0.90(0.60–1.34)	0.586	1.22(0.87–1.70)	0.252	**2.02(1.36–3.00)**	**0.001**	0.92(0.68–1.24)	0.577	1.34(0.67–2.71)	0.410
	Good	1.09(0.66–1.81)	0.729	1.13(0.74–1.74)	0.571	**2.83(1.80–4.47)**	**<0.001**	0.95(0.65–1.40)	0.802	1.44(0.61–3.43)	0.410

^a^ *CVD* Cardio-vascular diseases

^b^ *AOR* Adjusted odds ratio (adjusted for: age, gender, socio-economic status and family history)

^c^ *CI* Confidence intervals

Bolfaced figures indicate statistically significant results where *p* value was <0.05

Compared to females, male students were more likely to perform moderate/vigorous activities as were those who had better (compared to poor) knowledge regarding raised blood pressure [AOR respectively: 3.40(2.55–4.54) and 1.39(1.03–1.88)]. Students belonging to upper SES had lower odds of being involved in such activities compared to their counterparts belonging to lower SES [AOR = 0.59(0.37–0.94)].

Regarding duration of exercise, older subjects were less likely to be physically active for at least 1 h per day [AOR = 0.77(0.64–0.94)]. Compared to females, male students were physically more active [AOR = 7.77(4.61–13.07)]. Good knowledge regarding overall CVD, its risk factors and healthy eating was also associated with higher odds of being physically active for adequate duration [AORs respectively: 2.90(1.46–5.78), 2.06(1.09–3.90) and 2.23(1.12–4.40)] (Table 3).

**Table 3:** Association of socio-demographics, CVD ^a^-related knowledge and perception with exercise habits among participating adolescent school-students of Kolkata (*N* = 1652)

Sociodemographic factors, knowledge and perceptions			Exercise (Reference = No)					
			Moderate and Vigorous Activity		Exercise			
			Yes		Inadequate exercise		Adequate exercise	
Variables		Categories	AOR ^b^ (95%CI ^c^)	*p* value	AOR ^b^ (95%CI ^c^)	*p* value	AOR ^b^ (95%CI ^c^)	*p* value
Age in years			1.00(0.84–1.18)	0.956	**0.77(0.64–0.94)**	**0.009**	0.82(0.66–1.02)	0.072
Gender of student (Reference = Female)		Male	**3.40(2.55–4.54)**	**<0.001**	**2.63(1.61–4.29)**	**<0.001**	**7.77(4.61–13.07)**	**<0.001**
Socio-economic status (Reference = Lower)		Middle	0.96(0.65–1.42)	0.839	0.84(0.46–1.52)	0.557	0.81(0.42–1.56)	0.531
		Upper	**0.59(0.37–0.94)**	**0.026**	1.01(0.50–2.02)	0.981	0.58(0.27–1.25)	0.1634
Family history of death due to cardio-vascular diseases (Reference = No)		Yes	1.23(0.88–1.72)	0.225	0.98(0.60–1.60)	0.942	1.21(0.71–2.05)	0.490
Level of knowledge regarding	Cardio-vascular diseases (Reference = Poor)	Average	1.08(0.79–1.49)	0.637	0.88(0.56–1.39)	0.592	0.96(0.59–1.59)	0.885
		Good	1.12(0.79–1.59)	0.523	1.05(0.62–1.80)	0.852	1.17(0.65–2.10)	0.592
	Blood pressure and its abnormalities (Reference = Poor)	Average	**1.39(1.03–1.88)**	**0.031**	1.16(0.74–1.81)	0.514	1.57(0.97–2.55)	0.068
		Good	1.17(0.76–1.79)	0.479	1.73(0.82–3.65)	0.152	1.88(0.85–4.19)	0.121
	Risk factors for cardio-vascular diseases (Reference = Poor)	Average	1.05(0.76–1.44)	0.769	1.00(0.64–1.55)	0.992	1.04(0.64–1.70)	0.872
		Good	0.97(0.68–1.38)	0.851	**2.38(1.32–4.30)**	**0.004**	**2.06(1.09–3.90)**	**0.026**
	Prevention of cardio-vascular diseases (Reference = Poor)	Average	0.85(0.62–1.15)	0.295	1.42(0.91–2.22)	0.122	1.14(0.70–1.86)	0.603
		Good	1.04(0.70–1.55)	0.841	1.81(0.93–3.51)	0.079	1.74(0.86–3.54)	0.126
	Healthy nutritional habits (Reference = Poor)	Average	1.03(0.76–1.42)	0.832	**1.91(1.18–3.07)**	**0.008**	**1.79(1.07–3.01)**	**0.028**
		Good	0.94(0.64–1.37)	0.832	**2.71(1.44–5.09)**	**0.002**	**2.23(1.12–4.40)**	**0.022**
	Cardio-vascular diseases and their prevention (Overall) (Reference = Poor)	Average	0.87(0.64–1.19)	0.385	**2.55(1.62–4.01)**	**<0.001**	**1.91(1.16–3.15)**	**0.011**
		Good	1.18(0.81–1.72)	0.403	**2.89(1.52–5.52)**	**0.001**	**2.90(1.46–5.78)**	**0.002**
Perception	Indicate which of the following best describes your body size (Reference = Normal)	Underweight	0.94(0.63–1.39)	0.742	1.39(0.75–2.59)	0.295	1.25(0.64–2.45)	0.508
		Overweight	0.99(0.71–1.38)	0.958	1.22(0.75–1.98)	0.421	1.18(0.69–2.00)	0.553
	Do you feel that you might be at risk for future heart diseases? (Reference = No)	Yes	1.09(0.76–1.56)	0.631	1.28(0.74–2.21)	0.375	1.35(0.74–2.44)	0.328
	In general, how would you describe your health? (Reference = Good)	Average	0.86(0.64–1.17)	0.336	1.17(0.76–1.80)	0.471	0.98(0.61–1.57)	0.926
		Poor	1.22(0.62–2.37)	0.564	2.62(0.76–9.06)	0.128	2.81(0.76–10.43)	0.122

^a^ *CVD* Cardio-vascular diseases

^b^ *AOR* Adjusted odds ratio (adjusted for: age, gender, socio-economic status and family history)

^c^ *CI* Confidence interval

Bolfaced figures indicate statistically significant results where *p* value was <0.05

Increase in age was associated with intake of higher number of meals [AOR_3 meals_ = 1.27(1.08–1.51) and AOR > _3 meals_ = 1.27(1.01–1.59), reference = 1–2 meals]. Compared to females, males had higher frequency of major meals and snacking [AOR > _3 meals_ = 1.53(1.02–2.30), reference = 1/2 meals; AOR_4 times snacking_ = 1.61(1.23–2.10) and AOR > _4 times snacking_ = 1.68(1.04–2.71), reference = ≤3 times]. Male students were also less likely to skip meals [AOR = 0.62(0.42–0.93), reference = never]. With reference to those belonging to lower SES, affluent participants had higher likelihood of taking more meals/day [AOR_3 meals_ = 8.67(5.26–14.29) and AOR > _3 meals_ = 11.90(5.01–28.29), reference = 1/2 meals] and eating food outside. [AOR_sometimes_ = 2.78(1.18–6.53), reference = never] Regarding snacking habit, students belonging to higher SES were also likely to have less frequency [AOR_4 times_ = 0.57(0.37–0.87) and AOR > _4_ times =0.20(0.09–0.49), reference = never].

Participants who had better knowledge (reference poor) regarding CVDs in general, its risk factors, healthy eating habits and overall about CVD with its prevention were more likely to eat >3 major meals/day [respective AORs: 1.74(1.07–2.84), 2.17(1.34–3.53), 2.08(1.26–3.43) and 2.90(1.72–4.88)]. With the same reference, participants with good knowledge regarding healthy nutritional habits had higher odds of snacking [AOR = 1.55(1.09–2.22)].

Subjects who perceived themselves to be overweight were less likely to take higher number of meals per day [AOR_3 meals_ = 0.63(0.45–0.89) and AOR > _3 meals_ = 0.42(0.25–0.70), ref. = 1/2 meals] and to eat outside [AOR = 0.45(0.28–0.72)] relative to who perceived themselves as normal weight. Individuals who perceived themselves at risk for future CVDs were more likely to take >3 meals per day [AOR = 1.62(1.02–2.58)] and skip meals [AOR = 2.04(1.28–3.25)] than those who did not perceive themselves to be at risk (Table 4).

**Table 4:** Association of socio-demographics, CVD ^a^-related knowledge and perception with components of eating habits among participating adolescent school-students of Kolkata (*N* = 1652)

Socio-demographics, knowledge and perception			Meals taken/day (Reference = One/two)				Snacks taken/day? (Reference = Three times or less)				Skipping meals (Reference = Never)				Eating food in the street-shop/restaurant (Reference = Never)			
			Three		More than three		Four times		More than four times		Sometimes		Often		Sometimes		Often	
Variables		Categories	AOR ^b^ (95%CI ^c^)	*p* value	AOR ^b^ (95%CI ^c^)	*p* value	AOR ^b^ (95%CI ^c^)	*p* value	AOR ^b^ (95%CI ^c^)	*p* value	AOR ^b^ (95%CI ^c^)	*p* value	AOR ^b^ (95%CI ^c^)	*p* value	AOR ^b^ (95%CI ^c^)	*p* value	AOR ^b^ (95%CI ^c^)	*p* value
Age in years			**1.27(1.08–1.51)**	**0.004**	**1.27(1.01–1.59)**	**0.044**	0.90(0.77–1.05)	0.184	0.93(0.71–1.22)	0.616	1.05(0.89–1.23)	0.572	1.11(0.90–1.37)	0.328	1.20(0.90–1.59)	0.206	1.26(0.95–1.69)	0.113
Other socio-demographic factors	Gender of student (Reference = Female)	Male	1.05(0.79–1.41)	0.739	**1.53(1.02–2.30)**	**0.039**	**1.61(1.23–2.10)**	**<0.001**	**1.68(1.04–2.71)**	**0.034**	0.80(0.61–1.04)	0.099	**0.62(0.42–0.93)**	**0.021**	1.06(0.68–1.66)	0.790	1.32(0.83–2.10)	0.234
	Socio-economic status (Reference = Lower)	Middle	**1.91(1.24–2.93)**	**0.003**	**3.62(1.61–8.10)**	**0.002**	0.86(0.59–1.26)	0.447	0.74(0.41–1.34)	0.314	0.87(0.60–1.28)	0.482	0.77(0.45–1.33)	0.348	0.87(0.45–1.66)	0.667	**0.45(0.24–0.85)**	**0.015**
		Upper	**8.67(5.26–14.29)**	**<0.001**	**11.90(5.01–28.29)**	**<0.001**	**0.57(0.37–0.87)**	**0.010**	**0.20(0.09–0.49)**	**<0.001**	1.29(0.83–2.00)	0.257	1.06(0.56–1.98)	0.862	**2.78(1.18–6.53)**	**0.019**	1.04(0.44–2.47)	0.934
	Family history of death due to CVD (Reference = No)	Yes	0.92(0.65–1.30)	0.624	0.89(0.54–1.48)	0.657	1.06(0.77–1.46)	0.704	1.27(0.75–2.16)	0.374	1.20(0.87–1.66)	0.275	1.16(0.72–1.86)	0.541	0.94(0.56–1.56)	0.802	1.09(0.64–1.84)	0.750
Level of knowledge regarding	CVD (Reference = Poor)	Average	**1.46(1.05–2.01)**	**0.023**	1.42(0.89–2.27)	0.145	0.80(0.60–1.08)	0.146	1.16(0.68–1.98)	0.592	1.10(0.81–1.49)	0.536	1.15(0.74–1.78)	0.538	0.78(0.48–1.28)	0.330	0.67(0.41–1.12)	0.130
		Good	1.24(0.86–1.79)	0.245	**1.74(1.07–2.84)**	**0.027**	1.04(0.75–1.45)	0.799	1.25(0.68–2.29)	0.477	1.21(0.87–1.70)	0.256	1.02(0.61–1.69)	0.947	0.88(0.50–1.55)	0.658	0.76(0.42–1.36)	0.355
	Blood pressure and its abnormalities (Reference = Poor)	Average	**1.42(1.13–1.80)**	**0.003**	**1.53(1.07–2.18)**	**0.021**	0.91(0.72–1.15)	0.421	1.23(0.84–1.79)	0.289	1.14(0.90–1.43)	0.282	1.00(0.71–1.41)	1.000	1.35(0.91–1.99)	0.134	1.29(0.86–1.93)	0.219
		Good	1.12(0.71–1.77)	0.627	1.76(1.00–3.11)	0.051	0.94(0.63–1.41)	0.779	0.75(0.33–1.68)	0.482	1.19(0.78–1.80)	0.422	1.14(0.62–2.07)	0.675	0.53(0.28–1.01)	0.053	0.72(0.38–1.37)	0.317
	Risk factors for CVD (Reference = Poor)	Average	**1.51(1.09–2.09)**	**0.014**	1.18(0.73–1.90)	0.509	1.03(0.76–1.39)	0.867	1.26(0.75–2.14)	0.385	1.05(0.77–1.42)	0.780	0.92(0.59–1.42)	0.694	1.63(0.97–2.76)	0.067	**2.04(1.19–3.49)**	**0.009**
		Good	**1.93(1.34–2.78)**	**0.001**	**2.17(1.34–3.53)**	**0.002**	1.11(0.80–1.55)	0.530	0.93(0.49–1.76)	0.824	1.00(0.71–1.41)	0.984	0.80(0.48–1.33)	0.388	1.28(0.75–2.16)	0.365	0.95(0.54–1.67)	0.864
	Prevention of CVD (Reference = Poor)	Average	1.28(0.94–1.75)	0.118	1.41(0.91–2.17)	0.122	1.12(0.84–1.50)	0.430	1.37(0.81–2.31)	0.243	1.15(0.86–1.54)	0.341	0.83(0.54–1.29)	0.417	1.21(0.76–1.93)	0.412	0.91(0.56–1.48)	0.708
		Good	**1.81(1.20–2.74)**	**0.005**	1.64(0.93–2.89)	0.087	0.95(0.65–1.39)	0.787	1.64(0.85–3.19)	0.142	1.05(0.71–1.54)	0.812	0.93(0.53–1.63)	0.788	2.06(0.96–4.39)	0.062	1.59(0.73–3.48)	0.243
	Healthy nutritional habits (Reference = Poor)	Average	1.32(0.96–1.82)	0.086	1.18(0.75–1.88)	0.476	1.22(0.91–1.64)	0.189	1.03(0.60–1.78)	0.912	1.13(0.84–1.53)	0.427	0.91(0.58–1.42)	0.671	1.23(0.75–2.01)	0.419	1.22(0.73–2.04)	0.445
		Good	1.46(0.99–2.16)	0.058	**2.08(1.26–3.43)**	**0.004**	**1.55(1.09–2.22)**	**0.015**	1.35(0.71–2.57)	0.355	1.07(0.75–1.53)	0.709	0.85(0.49–1.45)	0.546	1.43(0.76–2.66)	0.266	1.28(0.67–2.45)	0.455
	CVD and their prevention (Overall) (Reference = Poor)	Average	**1.57(1.14–2.15)**	**0.006**	1.14(0.72–1.82)	0.583	0.98(0.73–1.31)	0.888	0.91(0.54–1.54)	0.736	1.20(0.89–1.61)	0.237	1.00(0.65–1.54)	0.998	1.29(0.81–2.07)	0.287	1.08(0.66–1.76)	0.762
		Good	**2.36(1.57–3.54)**	**<0.001**	**2.90(1.72–4.88)**	**<0.001**	1.29(0.89–1.85)	0.177	1.17(0.60–2.27)	0.652	1.09(0.75–1.58)	0.653	0.74(0.42–1.31)	0.297	1.48(0.77–2.82)	0.239	1.33(0.68–2.60)	0.399
Perception regarding	Own body size (Reference = Normal)	Underweight	1.03(0.69–1.53)	0.890	0.54(0.28–1.03)	0.063	1.28(0.88–1.85)	0.192	0.92(0.47–1.79)	0.800	0.96(0.66–1.40)	0.827	1.12(0.64–1.94)	0.699	0.94(0.48–1.85)	0.866	1.28(0.65–2.54)	0.472
		Overweight	**0.63(0.45–0.89)**	**0.008**	**0.42(0.25–0.70)**	**0.001**	1.18(0.87–1.61)	0.292	0.93(0.52–1.65)	0.802	0.94(0.68–1.29)	0.688	1.24(0.79–1.96)	0.346	**0.45(0.28–0.72)**	**0.001**	**0.55(0.33–0.89)**	**0.016**
	Future risk of CVDs (Reference = No)	Yes	0.93(0.64–1.35)	0.698	**1.62(1.02–2.58)**	**0.042**	0.94(0.67–1.32)	0.721	1.16(0.65–2.08)	0.615	1.23(0.87–1.75)	0.249	**2.04(1.28–3.25)**	**0.003**	0.88(0.51–1.51)	0.631	1.01(0.57–1.77)	0.978
	Own overall health (Reference = Good)	Average	0.89(0.66–1.21)	0.470	1.16(0.76–1.76)	0.496	1.19(0.90–1.57)	0.225	1.19(0.72–1.97)	0.498	1.10(0.83–1.46)	0.513	1.21(0.80–1.83)	0.376	1.02(0.63–1.63)	0.945	1.25(0.77–2.03)	0.371
		Poor	0.81(0.40–1.63)	0.552	1.05(0.40–2.72)	0.925	1.25(0.65–2.42)	0.503	2.45(0.96–6.23)	0.061	1.57(0.80–3.09)	0.190	1.82(0.74–4.48)	0.195	**0.38(0.16–0.90)**	**0.027**	0.54(0.23–1.30)	0.172

^a^ *CVD* Cardio-vascular diseases

^b^ *AOR* Adjusted odds ratio (adjusted for: age, gender, socio-economic status and family history)

^c^ *CI* Confidence interval

Bolfaced figures indicate statistically significant results where *p* value was <0.05

Overall frequency of eating increased with age [AOR > _appropriate_ = 1.24(1.05–1.47), reference = 5 times]. Considering major meals and snacking together, participants with good knowledge regarding risk factors [AOR = 1.84(1.17–2.91)], healthy nutritional habits [AOR = 1.69(1.05–2.71)] as well as overall good knowledge [AOR = 2.19(1.34–3.58)] were more likely to take food >5 times/day than those with poor knowledge.

Older students [AOR_average_ = 0.82(0.69–0.98) and AOR_good_ = 0.70(0.56–0.89)], those from higher SES [AOR = 0.23(0.11–0.47)], who had overall good knowledge regarding CVDs and their prevention [AOR = 0.55(0.32–0.94)] and perceived themselves to be at higher risk for future CVDs [AOR = 0.54(0.36–0.80)] were less likely to practice healthy eating behaviors. (Table 5).

**Table 5:** Association of socio-demographics, CVD ^a^-related knowledge and perception with overall frequency and habit regarding eating among participating adolescent school-students of Kolkata (*N* = 1652)

Socio-demographics, knowledge and perception			Average frequency of food intake (Reference = Approximately appropriate)				Overall eating habit (Reference = Poor)			
			More than appropriate		Much more than appropriate		Average		Good	
Variables		Categories	AOR ^b^ (95%CI ^c^)	*p* value	AOR ^b^ (95%CI ^c^)	*p* value	AOR ^b^ (95%CI ^c^)	*p* value	AOR ^b^ (95%CI ^c^)	*p* value
Age in years			**1.24(1.05–1.47)**	**0.011**	1.23(1.00–1.53)	0.054	**0.82(0.69–0.98)**	**0.031**	**0.70(0.56–0.89)**	**0.003**
	Gender of student (Reference = Female)	Male	1.04(0.78–1.39)	0.791	1.35(0.93–1.97)	0.116	1.17(0.87–1.56)	0.302	0.88(0.61–1.26)	0.480
	Socio-economic status (Reference = Lower)	Middle	1.44(0.96–2.14)	0.077	**2.58(1.35–4.91)**	**0.004**	0.89(0.60–1.33)	0.576	1.45(0.89–2.36)	0.135
		Upper	**5.88(3.65–9.48)**	**<0.001**	**6.75(3.28–13.89)**	**<0.001**	0.67(0.42–1.06)	0.083	**0.23(0.11–0.47)**	**<0.001**
	Family history of CVD death (Reference = No)	Yes	1.01(0.72–1.41)	0.973	0.99(0.63–1.56)	0.957	1.10(0.78–1.55)	0.594	0.79(0.52–1.21)	0.283
Level of knowledge regarding	CVD (Reference = Poor)	Average	**1.52(1.10–2.09)**	**0.011**	1.46(0.95–2.24)	0.082	0.79(0.57–1.10)	0.158	0.93(0.63–1.37)	0.703
		Good	1.25(0.87–1.78)	0.234	1.47(0.93–2.34)	0.099	0.85(0.60–1.22)	0.377	0.90(0.58–1.42)	0.658
	Blood pressure and its abnormalities (Reference = Poor)	Average	1.32(0.97–1.80)	0.073	1.41(0.94–2.11)	0.093	0.91(0.67–1.24)	0.546	**0.63(0.42–0.93)**	**0.021**
		Good	1.03(0.65–1.62)	0.906	1.56(0.91–2.69)	0.107	**0.62(0.39–1.00)**	**0.048**	0.85(0.50–1.46)	0.556
	Risk factors for CVD (Reference = Poor)	Average	**1.49(1.08–2.05)**	**0.017**	1.23(0.79–1.90)	0.357	0.96(0.70–1.33)	0.822	**0.62(0.41–0.93)**	**0.020**
		Good	**1.71(1.19–2.47)**	**0.004**	**1.84(1.17–2.91)**	**0.009**	0.78(0.54–1.13)	0.192	0.77(0.50–1.19)	0.238
	Prevention of CVD (Reference = Poor)	Average	1.26(0.92–1.72)	0.146	1.32(0.88–1.97)	0.175	0.99(0.72–1.36)	0.946	0.86(0.59–1.26)	0.446
		Good	**1.83(1.21–2.77)**	**0.004**	1.46(0.85–2.52)	0.169	1.10(0.73–1.65)	0.640	0.73(0.42–1.28)	0.274
	Healthy nutritional habits (Reference = Poor)	Average	1.27(0.93–1.75)	0.135	1.13(0.74–1.73)	0.5645	0.98(0.71–1.36)	0.921	0.77(0.51–1.14)	0.186
		Good	1.29(0.87–1.90)	0.202	**1.69(1.05–2.71)**	**0.032**	0.96(0.65–1.41)	0.835	0.70(0.43–1.16)	0.166
	CVD and their prevention (Overall) (Reference = Poor)	Average	**1.38(1.01–1.88)**	**0.046**	0.97(0.63–1.48)	0.878	0.89(0.65–1.23)	0.480	0.90(0.62–1.30)	0.564
		Good	**2.09(1.39–3.14)**	**<0.001**	**2.19(1.34–3.58)**	**0.002**	0.80(0.54–1.18)	0.259	**0.55(0.32–0.94)**	**0.028**
Perception about	Own body size (Reference = Normal)	Underweight	0.93(0.63–1.38)	0.721	0.66(0.38–1.16)	0.147	0.97(0.65–1.46)	0.890	0.83(0.50–1.36)	0.456
		Overweight	**0.61(0.44–0.86)**	**0.004**	**0.57(0.36–0.90)**	**0.015**	1.23(0.87–1.72)	0.240	1.23(0.82–1.85)	0.320
	Own risk for future CVD (Reference = No)	Yes	0.86(0.59–1.25)	0.434	**1.55(1.00–2.40)**	**0.048**	**0.54(0.36–0.80)**	**0.002**	0.65(0.41–1.03)	0.067
	Overall health (Reference = Good)	Average	0.87(0.64–1.17)	0.353	1.12(0.76–1.66)	0.578	0.85(0.62–1.15)	0.287	0.84(0.58–1.22)	0.367
		Poor	0.78(0.38–1.61)	0.504	1.73(0.79–3.82)	0.173	0.63(0.30–1.33)	0.226	0.82(0.35–1.89)	0.634

^a^ *CVD* Cardio-vascular diseases

^b^ *AOR* Adjusted odds ratio (adjusted for: age, gender, socio-economic status and family history)

^c^ *CI* Confidence interval

Bolfaced figures indicate statistically significant results where *p* value was <0.05

## Discussion

Current distribution reveals that persons belonging to 10–24 years age-group (1.8 billion) constitute the largest component of the global population and 1.5 billion of them hail from resource-poor settings [25, 35]. Adoption of healthy behaviors during adolescence is expected to serve as the foundation for ensuring longer life expectancy for this population group. Adolescence is considered as the period of vulnerability as well as the optimum opportunity to modify health-related risk behaviors [35]. On the contrary, young adults are always considered to be healthy and global health planners mostly neglect their health needs. Although some standardized health indicators are available for young in Western countries, such indicators are almost nonexistent in developing world. Moreover, adolescents’ risk perceptions in relation to health-related behaviors appears to be crucial in determining long-term health consequences [36–41]. Thus, educating adolescents regarding negative impacts of risk-taking and encouraging them to take responsibility of their own health seemed crucial in controlling adolescent health situation in countries like India.

### Knowledge regarding CVDs and their prevention

In this relatively large cross-sectional study, involving adolescent school-students of a metro city (Kolkata) in India, overall knowledge regarding CVDs and their prevention was observed to be poor among participants. The level of knowledge varied considerably across domains. About 60% students had average/good knowledge regarding CVDs in general. Similar findings were also reported from other states in India [42, 43] In our study about 23% of participants had good knowledge regarding risk factors for CVDs. Knowledge regarding lifestyle risk factors of NCDs was observed to be even lower among students in Kerala [44], as well as in Michigan [21] and Arizona [45] in US but relatively higher in Nepal [46]. A significant proportion of students in Pune reported obesity, physical inactivity and smoking as predictors of CVDs but could not identify other potential risk factors like serum cholesterol and hypertension [42]. Innovative and friendly educational intervention strategies should be developed for youth so that the gaps in knowledge about CVDs could be addressed successfully.

### Perceptions

Understanding the risk-associated thoughts and response was found to be fundamental in shaping perceptions to promote healthy behaviors. Slovic suggested that unfamiliar risks (new/unknown) were mostly avoided by people whereas familiar risks if perceived as controllable or self-chosen culminated into reduction of importance [47]. In addition, body image perception of adolescents and resultant dissatisfaction might negatively affect adolescent health behaviors [48, 49]. In our study, only 23.7% perceived themselves as overweight and they demonstrated relatively healthy eating habits. Care should also be taken to ensure that youth perception of overweight does not culminate into depression and harmful weight control practices.

About 82% of the participating adolescents did not perceive themselves to be at risk for future CVDs and even those who perceived the risk showed poor dietary practices. Similar findings were also reported from Nepal and Michigan (US) [21, 46]. One of the probable explanations might be that adolescents considered CVDs to be old age related problem and underestimated their future risks. We also observed that subjects with average/good knowledge regarding CVDs were more likely (compared to those with poor knowledge) to perceive themselves at risk for future CVDs. Promotion of school-based cardiovascular health programs might be crucial in dispelling myths and misconceptions with eventual prevention of early onset atherosclerotic changes in arterial walls.

### Positive family history

Consistent with previous studies [43], little above one-fifth participants had positive family history of CVDs while in Kerala about 2.9% had such history [44]. Early screening of these at-risk children and appropriate interventions might be effective in prevention of early onset CVDs.

### Physical activity

Less than 30% subjects reported to get engage in moderate-to-vigorous exercises regularly which was lower than that reported among school-aged children in Delhi [43] and in Gujarat [50] but higher than in Kerala [44]. Inadequate physical activity was also reported from Ontario, US [51] and in Uttarakhand, Maharashtra, Madhya Pradesh and Andhra Pradesh in India [52]. Consistent with previous research in Brazil [53] and Taiwan [35], male students were more likely to be physically active in our study. Previous studies suggested a probable decline in physical activity during transition from adolescence into adulthood, especially between 15 and 23 years [54, 55]. One of the probable reasons might be that as adolescents get older, they lack self-motivation and become less compliant to healthy advices. On the other hand we observed that students with good/average knowledge regarding CVDs were somewhat physically active. Based on this observation, it seemed reasonable to hypothesize that adolescents who were well informed about negative health effects of physical inactivity were possibly more motivated to be physically active.

### Eating habits

More than half of the participants of the current study demonstrated poor eating-behaviors. Older subjects, males and those who were economically better-placed, took more than appropriate number of meals (major meals & snacks). Similar observations were also reported among adolescents from Arab [56], Europe [57], China [58] and other states in India: Baroda [31], Mysore [30], Uttarakhand, Maharashtra, Kerala, Madhya Pradesh and Andhra Pradesh [52]. There were huge gaps between knowledge about CVDs and eating behavior among study participants. Subjects with good knowledge were likely to take higher number of meals and snacks per day. This observation might be explained by possible influence of frequency and pattern of eating in family. Friendly interactions between adolescents and their parents/teachers/nutritionists at regular interval might be effective in shaping their eating habits.

Although higher physical inactivity has often been linked with increased risk of morbidity and mortality, an estimated 60% of population in the world do not perform recommended exercise regularly. As per WHO estimates, 80% of premature heart diseases and 80% of diabetes could be prevented by interplay of healthy diet, physical activity and tobacco avoidance [35]. Moreover, analysis of Northern Finland birth cohort data revealed that risk of obesity was significantly lower in adolescents who ate five meals/day [32] while intake of inappropriate number of meals/day was associated with future development of obesity [59]. Thus, early identification of these unattended risk factors through screening, timely interventions and raising awareness about healthy lifestyle during adolescence might be more effective in controlling CVD epidemics in India. Furthermore, lifestyle modifications at younger age might be more successful than changing acquired harmful habits in adults. Thus, a concerted public health effort towards modifying food and physical activity environments in schools and in communities seemed to be the need of the hour.

### Study limitations

There were some major limitations in the present study. Alike any other observational study associations observed here, should not be interpreted as causal owing to the potentials for residual confounding and other systematic errors. Because of the cross-sectional design, potentials for temporal ambiguity should also be kept in mind. Non-response might have affected the representativeness of the study population. Thus, efforts for extrapolation of results beyond the study sample to all adolescent urban students in the study area need some caution. But we still believe that the observations were quite generalizable, owing to the robust sampling strategy and good response rate. Self-reported history of dietary pattern and exercise habits were also likely to suffer from some social desirability bias and issues of recall, although we considered them to be non-differential. Moreover, to minimize the chances of information bias, we urged the subjects to recall for only a short period of 1 week. Despite these limitations, by virtue of large sample size, robust methodology and advanced statistical analyses, we believe that the results of this research will be useful in understanding the scenario pertaining to CVD related knowledge, perceptions regarding related risk, exercise habit, dietary practices and interplays thereof among adolescents of Kolkata, India.

## Conclusions

Eating and exercise habits were found to be quite poor among large proportion of adolescent school-students of Kolkata. Lack of adequate knowledge and poor perception about CVD, related risk and importance of their prevention were potential contributors. Also, there existed a large gap between CVD-related knowledge and eating and exercise related practice. As such, our results suggest that there is a critical need for not only the improvement of knowledge and awareness regarding CVD and its related risks, but also actions that will help to bridge the knowledge-practice gap for eating and exercise habits. Given childhood origin of CVDs and possibility of reversing/slowing these atherosclerotic changes through early life style modifications, youth friendly, multicomponent cardiovascular health promotion programs are urgently required to raise awareness and appropriate translation of the awareness in to healthy practices in this target population.

## Abbreviations

95%CI: 95% confidence interval
AOR: Adjusted odds ratio
CVD: Cardiovascular disease(s)
IHD: Ischemic heart disease(s)
NCD: Non-communicable disease(s)
OR: Odds ratio
SES: Socio-economic status
